# Clinical study of safety and immunogenicity of pentavalent DTP-HB-Hib vaccine administered by disposable-syringe jet injector in India

**DOI:** 10.1016/j.conctc.2019.100321

**Published:** 2019-01-09

**Authors:** Ashish Bavdekar, Nandini Malshe, Latha Ravichandran, Amita Sapru, Anand Kawade, Sanjay Lalwani, Sonali Palkar, Neeta Hanumante, Bhagwat Gunale, Dhananjay Kapse, Amol Chaudhari, Tara Miller, Laura Saganic, Courtney Jarrahian, Sarah McGray, Darin Zehrung, Prasad S. Kulkarni

**Affiliations:** a KEM Hospital Research Centre, Pune, India; b Bharati Vidyapeeth Deemed University Medical College, Pune, India; c Sri Ramachandra Medical Centre, Chennai, India; d Serum Institute of India Pvt. Ltd., Pune, India; e PharmaJet, Golden, CO, USA; f PATH, Seattle, WA, USA

**Keywords:** DTP-HB-Hib vaccine, Jet injector, DSJI, Vaccine adjuvant, Immunogenicity, Injection site reactions

## Abstract

**Introduction:**

We conducted a randomized, observer-blind, non-inferiority, parallel-group clinical study of diphtheria, tetanus, pertussis, hepatitis B, and *Haemophilus influenzae* type b conjugate (pentavalent) vaccination of infants in India. Goals were to determine whether the seropositivity rate after vaccination via disposable-syringe jet injector (DSJI) was non-inferior to that via needle and syringe (N-S), and to compare the safety of vaccination by the two methods.

**Methods:**

Healthy children received a three-dose series of vaccine intramuscularly by DSJI or N-S beginning at 6–8 weeks of age. Immunoglobulin G antibody levels were measured by ELISA at 4–6 weeks after the third dose. The main secondary endpoint was safety, measured as injection site and systemic reactions.

**Discussion:**

The study was stopped early out of caution beyond that specified in the protocol stopping criteria, after the Data Safety Committee noted a higher frequency of injection site reactions, especially moderate and severe, in the DSJI group. As a result, 128 subjects—DSJI group 61; N-S group 67—completed the study, rather than the 340 planned, and the study was not sufficiently powered to compare immunogenicity endpoints for the groups. Descriptive statistics indicate that seropositivity induced by vaccination with the DSJI was similar to that of N-S for all five antigens. Pentavalent vaccine includes whole-cell pertussis vaccine and an aluminum adjuvant, which may have contributed to the higher number of local reactions with the DSJI. The reactions caused no serious or long-term sequelae, and may be more acceptable in other populations or circumstances.

US National Institutes of Health clinical trials identifier: NCT02409095.

## Abbreviations

DSJI: disposable-syringe jet injector
DTP-HB-Hib: diphtheria, tetanus, pertussis-hepatitis B-*Haemophilus influenzae* type b
ELISA: enzyme-linked immunosorbent assay
GMT: geometric mean titer
Hib: hepatitis B
IgG: immunoglobulin G
MMR: measles-mumps-rubella
N-S: needle and syringe
PRP: polyribosyl-ribitol-phosphate
SAE: serious adverse event

## 1 Introduction

The World Health Organization recommends universal immunization with 12 vaccine antigens, including diphtheria, tetanus, pertussis, hepatitis B, and *Haemophilus influenzae* type b (Hib) [1]. These five antigens are available as a pentavalent combination DTP-HB-Hib vaccine, which is used in the national immunization programs of many developing countries [2], including India [3]. Worldwide, more than 20 million children are under-immunized for the recommended vaccines, nearly one-third of whom live in India [4].

Vaccines are typically administered as injections with autodisable needle and syringe (N-S); however, this can result in needlestick injuries and cross-infection, and can create dangerous sharps waste in communities [5]. An alternative technology, jet injection, uses high pressure to deliver a fine stream of fluid that penetrates the skin to deposit vaccine in dermal, subcutaneous, or muscle tissue, as required. In the 1950s, multiuse-nozzle jet injectors made high-throughput mass vaccination campaigns possible for such diseases as smallpox, polio, meningitis, and measles [5]. The potential benefits of jet injectors include providing more consistent delivery of vaccines, reducing wastage, eliminating transport of large volumes of sharps, and reducing the risk of needlestick injuries and costs associated with sharps waste. However, the multiuse injectors were withdrawn from public health use in the 1990s because of the risk of cross-contamination [5].

Disposable-syringe jet injectors (DSJIs) use a sterile, single-dose, disposable syringe for each patient, avoiding the cross-contamination risks of multiuse-nozzle injectors. Most remaining risks for DSJIs also apply to vaccination by N-S, such as pain, bleeding, or swelling at the injection site; or user error in administering the dose to the correct tissue layer. DSJIs can be used to deliver liquid and reconstituted lyophilized vaccines from single-dose or multidose vial and ampoule presentations. The efficacy and safety of vaccines for a number of diseases—hepatitis A and B, Hib, polio, tetanus, typhoid, and yellow fever, among others—have been shown to be similar whether administered by jet injection or N-S [5].

We conducted a phase IV, randomized, observer-blind, non-inferiority, parallel-group clinical study of pentavalent vaccination in infants in India. The goals of the study were to determine whether the seropositivity rate for recipients of pentavalent vaccine administered using a DSJI was non-inferior to that of subjects administered vaccine via N-S, for all components of the vaccine, and to compare the safety of vaccination by the two methods. A result of non-inferiority for the DSJI would support use of a DSJI for pentavalent vaccination.

## 2 Methods and materials

### 2.1 Vaccine and injection devices

The investigational product for this study was the liquid pentavalent vaccine from the Serum Institute of India Pvt. Ltd. (Pune, India), administered by the Stratis^®^ DSJI (PharmaJet, Golden, CO, USA). The vaccine was diphtheria, tetanus, pertussis (whole cell), hepatitis B (rDNA), and *Haemophilus influenzae* type b conjugate vaccine (adsorbed), batch number 137L4011E. Each 0.5 mL dose contained the following antigens: diphtheria toxoid ≤25 Lf (≥30 IU); tetanus toxoid ≥2.5 Lf (≥40 IU); *Bordetella pertussis* (whole cell) ≤16 OU (≥4 IU); recombinant hepatitis B virus surface antigen (rDNA) ≥10 μg; and Hib conjugate vaccine (purified capsular polysaccharide, polyribosyl-ribitol-phosphate [PRP] conjugated to tetanus toxoid) 10 μg. The vaccine also contained the adjuvant aluminum phosphate, ≤1.25 mg, and the preservative thiomersal, 0.005%. The Stratis device used in this study has regulatory clearance in the United States [6] and the European Economic Area [7], and is prequalified by the World Health Organization [8]. The batch numbers for the Stratis device were 25854275 and 23436455. The reference product was the same batch of vaccine from the Serum Institute of India administered via N-S. The pentavalent vaccine was administered as a three-dose series of 0.5 mL each by deep intramuscular injection in the anterolateral aspect of the thigh with either a DSJI or by N-S, starting at study day 0 (approximately 6–8 weeks of age) and repeated on study days 28 and 56.

### 2.2 Study populations and settings

The study was conducted at four sites across India between January and December 2015. Eligible participants were healthy infants aged 6–8 weeks. Excluded were children participating in other clinical trials; those with a history of diphtheria, tetanus, pertussis, and hepatitis B, or Hib, or of vaccination against these diseases; those with an acute or chronic clinically significant abnormality or illness; and those with a history of allergy to any of the vaccine components.

### 2.3 Randomization and blinding

A block randomization scheme allocated eligible subjects in a 1:1 ratio to pentavalent vaccine by either DSJI or N-S. Each block consisted of six subjects. The randomization list was generated using SAS^®^ version 9.2 (SAS Institute Inc., Cary, NC, USA). The list of randomization numbers and the group allocations covered with scratch labels were provided to all sites. The vaccinator allocated subjects to groups by scratching off the label corresponding to the randomization number on the list, just before vaccine administration. Investigator site personnel—except for staff administering the vaccine—and laboratory staff analyzing serum samples were not aware of which device was used for vaccination.

### 2.4 Immunogenicity evaluations

The immunogenicity evaluations were performed at Quest Diagnostics India Pvt. Ltd. (Gurgaon, India). Blood samples were collected from each subject on day 0 and at 4–6 weeks after the third dose. Serum samples were tested in pairs using commercially available enzyme-linked immunosorbent assay kits for diphtheria, pertussis, and tetanus immunoglobulin G (IgG) antibodies (Institut Virion/Serion GmbH, Wurzburg, Germany), for hepatitis B IgG antibodies (Ortho-Clinical Diagnostics, Raritan, NJ, USA), and for Hib antibodies (The Binding Site Group Ltd., Birmingham, United Kingdom). All serum samples were tested in duplicate and the mean of the two values was used for calculations.

### 2.5 Safety evaluations

At each visit, the subjects were physically examined and had a history taken for adverse events and concomitant medications. Parents were instructed to record solicited local and systemic reactions daily in a diary until 4 days after each vaccination. Any unsolicited adverse events and serious adverse events (SAEs) were also recorded throughout the study.

For all local reactions other than pain, the following criteria were used to classify the severity of the reactions: *mild—*the longest diameter was <20 mm; *moderate—*the longest diameter was ≥20 mm and ≤50 mm; and, *severe—*the longest diameter was >50 mm. Data were collected by parents (who had been trained by study staff) using a measuring scale (15 cm). The severity grading for measurable adverse events like redness, induration, bruising and swelling was done at the data management level as per criteria defined above. The severity grade for fever was defined as mild - 38 °C to <39 °C, moderate - 39 °C to <40 °C and severe - ≥ 40 °C. For pain and other systemic reactions (not measurable), the grading mild, moderate and severe was used as per investigator's clinical judgment.

### 2.6 Statistical analyses

The sample size was determined under the assumption that 90% of individuals in the control group would become seropositive, and that 10% of all subjects would withdraw from the study. The planned enrollment was 340 subjects in order to provide at least 80% power to reject the null hypothesis of inferiority, using a one-sided significance level of 0.025. The primary endpoint was seroprotection/seropositivity for each component 4–6 weeks after administration of the third dose. Seroprotection was defined as IgG antibody concentration ≥0.1 IU/mL (diphtheria and tetanus), ≥10 mIU/mL (hepatitis B), and ≥0.15 μg/mL for short-term protection and ≥1.0 μg/mL for long-term protection (Hib). As there is no correlate of protection for pertussis, seropositivity was defined as >50 IU/mL as per the kit instructions.

For secondary efficacy analyses, the geometric mean titers (GMTs) of anti-diphtheria, anti-tetanus toxoid, anti-pertussis, anti-hepatitis B, and anti-PRP antibodies were compared within each group between days 0 and 84. We also noted the seropositivity within each study group between days 0 and 84.

The other secondary endpoints were solicited local or systemic reactions within 4 days following the administration of each dose, and occurrence of any unsolicited adverse events and any SAEs throughout study participation.

The immunogenicity analyses are presented for the per-protocol population, which consisted of all subjects who had no major protocol violations and who completed all three vaccinations, with evaluable blood samples at days 0 and 84. Safety analyses are presented for the intention-to-treat population, constituting all subjects who received at least one dose of vaccine.

### 2.7 Ethical conduct of the study

Approvals for the study were obtained from the Drug Controller General of India, the institutional ethics committees of all study centers, and the Western Institutional Review Board. Subjects were screened for eligibility after written informed consent from their parents. The consent process was audio-visually recorded as per the prevalent regulatory norms.

## 3 Results

**Fig. 1:**
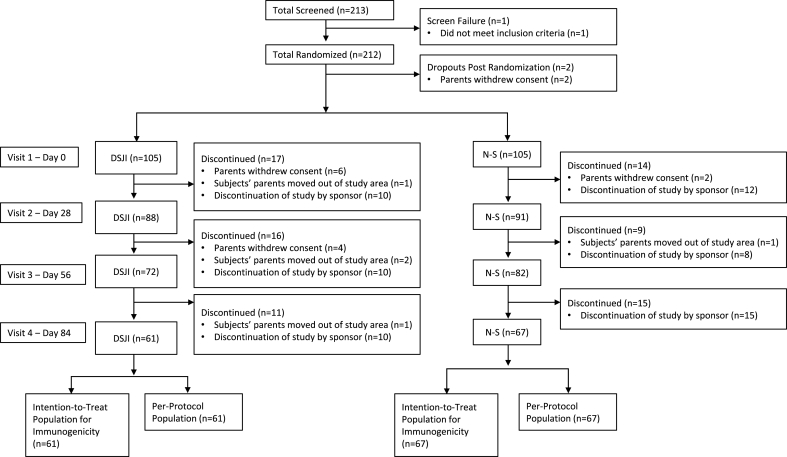
Study flowchart. Abbreviations: DSJI, disposable-syringe jet injector; N-S, needle and syringe.

A total population of 340 was planned for this study, but the study was terminated early. A total of 212 subjects were randomized at the time of study discontinuation. Two subjects were withdrawn before receiving study treatment; thus, 105 infants in each group received at least one dose of vaccine (Fig. 1). Only 128 subjects (61 in the DSJI group and 67 in the N-S group) completed the study per the protocol (per-protocol population), and the study was not sufficiently powered to compare the two groups for the immunogenicity endpoints. Thus, no formal statistical comparisons have been done, and results are presented descriptively.

At baseline, the DSJI and N-S groups appeared similar in age, height, and weight (Table 1). Nearly 60% of the subjects in the N-S group were females, compared with 53% in the DSJI group. The study was planned for 170 infants in each group but was stopped after 105 had been enrolled per group, because of the high frequency of injection site reactions in the DSJI group.

**Table 1:** Baseline characteristics: intention-to-treat population.^a^

Characteristic^b^	Disposable-syringe jet injector (n = 105)	Needle and syringe (n = 105)
Age (weeks)		
Mean (standard deviation)	6.9 (0.7)	6.8 (0.6)
Range	6.0–8.9	6.0–8.6
Height (cm)		
Mean (standard deviation)	55.1 (2.4)	54.8 (2.5)
Range	50.0–61.0	48.0–66.0
Weight (kg)		
Mean (standard deviation)	4.4 (0.6)	4.3 (0.5)
Range	3.3–6.3	2.9–5.6
Sex		
Male, n (%)	49 (46.7%)	43 (41.0%)
Female, n (%)	56 (53.3%)	62 (59.0%)

a The intention-to-treat population consisted of all participants who received at least one dose of study vaccine.

b All subjects were of Indian ethnicity.

The proportion of subjects who had at least one solicited local reaction with one or more doses of the study vaccines was 98% in the DSJI group and 99% in the N-S group. A total of 1480 solicited local reactions of pain, redness, swelling, induration, and bruising were reported across both groups for all three doses combined—868 in the DSJI group and 612 in the N-S group (Table 2). These reactions started within a minimum of 1 day and a maximum of 4 days from vaccine administration and lasted for a minimum of 1 day and a maximum of 101 days, with a mean of 3.1 days. Most of the solicited local reactions were mild or moderate in intensity, with 61 reactions of severe intensity across both groups (Table 2). There were more reactions of moderate and severe intensity in the DSJI than the N-S group, 299 versus 157 and 40 versus 21, respectively. All the solicited local reactions resolved without any sequelae.

**Table 2:** Solicited local reactions by intensity: intention-to-treat population, all three doses combined.

Local reaction	Disposable-syringe jet injector (n = 104)^a^ Subjects (Events)^b^				Needle and syringe (n = 104)^a^ Subjects (Events)^b^			
	All reactions	Mild	Moderate	Severe	All reactions	Mild	Moderate	Severe
Pain	102 (253)	79 (138)	52 (82)	22 (32)	103 (254)	87 (166)	50 (71)	15 (17)
Redness	88 (208)	78 (148)	45 (58)	1 (1)	68 (135)	61 (110)	19 (24)	1 (1)
Swelling	90 (186)	68 (105)	48 (73)	3 (5)	69 (127)	56 (92)	22 (33)	2 (2)
Induration	92 (207)	71 (119)	50 (83)	1 (2)	53 (93)	44 (63)	20 (29)	1 (1)
Bruising	14 (14)	11 (11)	3 (3)	0	3 (3)	3 (3)	0	0
At least one local reaction	102 (868)^c^	100 (521)	80 (299)	23 (40)	103 (612)	100 (434)	59 (157)	16 (21)

a Data for one subject in each group were missing.

b Some subjects experienced a local reaction (event) after more than one of the three doses in the series. In addition, some subjects experienced reactions of different intensities after different doses in the series.

c Data on intensity of reaction were missing for 8 events in the DSJI group, so the total number of events by intensity is 860 rather than 868.

The proportions of solicited systemic reactions were similar across the DSJI and N-S groups. The most commonly reported solicited systemic reactions were irritability, persistent crying, fever, and loss of appetite in both groups (Table 3). Most of these reactions were of mild to moderate intensity except for 27 that were severe (13 in the DSJI group and 14 in the N-S group, 2% each). All solicited systemic reactions resolved without any sequelae. Most of the events started within 1–4 days after vaccine administration and lasted for a minimum of 1 day and a maximum of 13 days.

**Table 3:** Solicited systemic reactions: intention-to-treat population, all three doses combined.

Solicited systemic reaction	Disposable-syringe jet injector (n = 104)^a^ Subjects (Events)^b^	Needle and syringe (n = 104)^a^ Subjects (Events)^b^
Irritability	87 (190)	87 (182)
Fever	76 (155)	76 (150)
Persistent crying	68 (157)	68 (146)
Loss of appetite	39 (62)	34 (51)
Diarrhea	11 (13)	9 (9)
Vomiting	9 (14)	9 (11)
Drowsiness	4 (4)	6 (6)
At least one systemic reaction	95 (595)	97 (555)

a Data for one subject in each group were missing.

b Some subjects experienced a systemic reaction (event) after more than one of the three doses in the series.

Across both the DSJI and N-S groups, 293 unsolicited adverse events were reported in 210 subjects (224 in the DSJI group and 69 in the N-S group). Of these, 125 in the DSJI group were judged to be related to the investigational product. All of these events were observed at the injection site (mainly discoloration, injection site bleeding, and nodules). In the N-S group, 11 unsolicited adverse events (mainly injection site bleeding) were judged to be related to the reference product. Most of these events were of mild intensity. None of the events were severe and all resolved without sequelae. No SAEs were reported in the study.

Seropositivity rates for the DSJI and N-S groups in the per-protocol population at baseline and at day 84 post vaccination (4 weeks after the third dose of vaccine) appeared comparable, by descriptive statistics, for all vaccine components (Table 4). There was an apparent rise in seropositivity rates for all components of the vaccine, except tetanus, from baseline to day 84 within both groups (Table 4). The GMTs appeared comparable between the two groups at baseline and at day 84 post vaccination for each component, and there was a rise in GMTs for all components of the vaccine, except tetanus, from baseline to day 84 within both groups (data not shown).

**Table 4:** Seroprotection/seropositivity^a^ at days 0 and 84 after vaccination in the per-protocol population.^b^

Vaccine component	Day 0		Day 84	
	Disposable-syringe jet injector (n = 61)	Needle and syringe (n = 67)	Disposable-syringe jet injector (n = 61)	Needle and syringe (n = 67)
Diphtheria	4 (6.6%)	7 (10.4%)	61 (100.0%)	64 (95.5%)
Tetanus	61 (100.0%)	66 (98.5%)	61 (100.0%)	66 (98.5%)
Pertussis	3 (4.9%)	1 (1.5%)	36 (59.0%)	41 (61.2%)
Hepatitis B	9 (14.8%)	9 (13.4%)	60 (98.4%)	66 (98.5%)
*Haemophilus influenzae* type B ≥1.0 μg/mL (long-term protection)	21 (34.4%)	24 (35.8%)	56 (91.8%)	62 (92.5%)
*Haemophilus influenzae* type B ≥0.15 μg/mL (short-term protection)	48 (78.7%)	55 (82.1%)	61 (100.0%)	65 (97.0%)

Abbreviations: DSJI, disposable-syringe jet injector; N-S, needle and syringe.

a Definitions for seroprotection/seropositivity are included in the “Methods and materials” section.

b The per-protocol population consisted of all subjects who had no major protocol violations and who completed all three vaccinations, with evaluable blood samples at day 0 and day 84.

## 4 Discussion

This study aimed to compare the safety and immunogenicity of pentavalent vaccine administered by DSJI with that administered by N-S. However, the study ended early because of an increased frequency of local injection-site reactions in the DSJI group, resulting in a sample size that did not allow use of statistical analyses to compare the two groups for the immunogenicity endpoints. In ending the study early, the sponsor acted out of an abundance of caution beyond that specified by the stopping criteria laid out in the protocol, which stated that the potential safety reasons for discontinuing the trial would be if there were a significant number of severe adverse events that were determined to be related to the study product. However, the difference between the number of moderate injection site reactions between the two groups (299 versus 157) led to the early discontinuation of the study.

Using descriptive statistics, we observed that at baseline, seropositivity rates for all five components of the vaccine were comparable between the two groups, as were the rates 4 weeks after the third dose. GMTs also appeared comparable between the groups at both time points. From baseline to post vaccination, there was an apparent rise in seropositivity rates for all vaccine components, within both groups, except for tetanus. The national immunization program in India provides all pregnant women with two doses of tetanus toxoid during pregnancy; thus, transplacental transfer of maternal antibodies against tetanus resulted in high baseline tetanus antibody titers in study infants.

For the safety endpoints, we observed that systemic reactions after vaccination with the DSJI were comparable to those after vaccination with N-S. However, overall incidence of injection site reactions—and importantly, of moderate and severe reactions—was higher for infants vaccinated by DSJI.

A similar clinical study was conducted in tandem with this one, at the same study sites, comparing immunogenicity and safety of measles-mumps-rubella (MMR) vaccine administered with either the DSJI or N-S [9]. That study was completed per the protocol, and concluded that MMR vaccination via DSJI met all non-inferiority criteria; it was as immunogenic and safe as vaccination by N-S. The children vaccinated in the MMR study were older—and therefore had larger body mass—than those in the present study: 15–18 months of age versus 6–8 weeks of age at the start of the study. In addition, MMR vaccine is administered subcutaneously, while pentavalent vaccine requires deeper, intramuscular administration, and importantly, pentavalent vaccine includes whole-cell pertussis vaccine and an aluminum adjuvant associated with increased injection site reactions, while MMR vaccine does not. Some reports indicate that vaccines with whole-cell antigens, aluminum, or other irritating ingredients can result in more soreness, swelling, and redness when administered by jet injector than by N-S [5]. Increased reactions may be a result of residual vaccine material remaining along the track left by the stream of liquid penetrating through the dermal and subcutaneous layers before entering the intramuscular layer [5].

The observed local reactions caused no serious or long-term sequelae, and may be more acceptable in other populations or circumstances. When considering delivery of adjuvanted and other reactogenic vaccines, the programmatic benefits of DSJI—such as more consistent delivery of vaccines, reduced wastage, elimination of transport of large volumes of sharps, and reduced risk of needlestick injuries and costs associated with sharps waste—should be balanced with the potential for an increase in local reactions. The safety and efficacy of vaccines delivered with a DSJI should be assessed on a case-by-case basis.

## Conflicts of interest

BG, DK, AC, and PSK are employed by Serum Institute of India Pvt. Ltd., which manufactures the pentavalent vaccine that was used in the study. TM is employed by PharmaJet, which manufactures the devices used in the study.

## Funding

This work was funded in part by a grant from the Bill & Melinda Gates Foundation. The views expressed herein are solely those of the authors and do not necessarily reflect the views of the Gates Foundation.

## References

[1] World Health Organization (WHO). WHO recommendations for routine immunization - summary tables. Available at: http://www.who.int/immunization/policy/immunization_tables/en/. Accessed April 13, 2018.

[2] Pentavalent vaccine support page. Gavi, the Vaccine Alliance, website. Available at: http://www.gavi.org/support/nvs/pentavalent/. Accessed April 13, 2018.

[3] Ministry of Health & Family Welfare, Government of India. Routine immunization background note. Available at: https://mohfw.gov.in/right-information-rti/rti-act-for-ministry/departments-health-and-family-welfare/immunization. Accessed April 13, 2018.

[4] (November 1, 2014). Pentavalent Vaccine Introductions Represent Historic Milestone for Immunisation in India [press Release].

[5] Weniger B.G., Papania M.J., Plotkin S., Orenstein W., Offit P. (2013). Chapter 61: alternative vaccine delivery methods. Vaccines.

[6] US Food and Drug Administration (FDA). Special 510(k) premarket notification: PharmaJet®, Inc.: PharmaJet® Stratis needle-free injection system [FDA clearance]. Available at: http://www.accessdata.fda.gov/cdrh_docs/pdf11/k111517.pdf. Accessed April 13, 2018.

[7] PharmaJet's Stratis needle*less* injection device is safe, fast and easy page. PharmaJet website. http://pharmajet.com/product/.

[8] Category E008: auto-disable syringe for fixed-dose immunization. World health organization PQS catalogue. Available at: http://apps.who.int/immunization_standards/vaccine_quality/pqs_catalogue/LinkPDF.aspx?UniqueID=df0fd1e0-53d8-474b-98f9-4007403b0e9c&TipoDoc=DataSheet&ID=0. Accessed April 13, 2018.

[9] Bavdekar A., Oswal J., Ramanan P.V., Aundhkar C., Venugopal P., Kapse D. (2018). Immunogenicity and safety of measles-mumps-rubella vaccine delivered by disposable-syringe jet injector in India: a randomized, parallel group, non-inferiority trial. Vaccine.

